# Female Intrasexual Competition and Its Link to Menopausal Stage, Sex Hormone Levels, and Personality Characteristics

**DOI:** 10.3389/fgwh.2021.740894

**Published:** 2021-08-30

**Authors:** Serena Fiacco, Carla Arpagaus, Laura Mernone, Ulrike Ehlert

**Affiliations:** ^1^Chair of Clinical Psychology and Psychotherapy, Psychological Institute, University of Zurich, Zurich, Switzerland; ^2^University Research Priority Program Dynamics of Healthy Aging Research Priority Program, University of Zurich, Zurich, Switzerland; ^3^Stress, Health and Reproductive Research Program, Department of Psychiatry, University of North Carolina at Chapel Hill, Chapel Hill, NC, United States

**Keywords:** sex hormones, self-esteem, female intrasexual competition, big 5 personality factors, postmenopausal women, premenopausal women

## Abstract

**Background:** Female intrasexual competition (ISC) represents a unique form of social interaction. It describes behaviors primarily applied to enhance a woman's ability to outcompete other women. Previous research suggests that female ISC is influenced by personality characteristics and sex hormones. Although these factors most likely interact to predict female ISC, no previous study has investigated those factors in parallel in order to link theories from social psychology and biology. Women at the end of the reproductive lifespan represent the ideal study population, as they allow for a controlled hormonal environment.

**Materials and Methods:** Healthy pre- (*N* = 53) and postmenopausal (*N* = 56) women were classified according to the Stages of Reproductive Aging Workshop (STRAW+10) criteria. In the follicular phase (for premenopausal women) or on a random day (for postmenopausal women), questionnaires were administered to assess the general tendency to compete intrasexually and the tendency to compete on appearance, attention/interpersonal success, and competence. Additionally, personality characteristics (neuroticism, extraversion, openness, agreeableness, conscientiousness, and self-esteem) were assessed. On the same day, each subject provided an 8 a.m. saliva sample for estradiol, testosterone, progesterone, and dehydroepiandrosterone sulfate. *T*-tests tested for between-group differences and separate multiple linear regression models tested for an effect of continuous hormone levels and personality characteristics on ISC. Further models were run, testing for an interaction with menopausal stage.

**Results:** No group differences in ISC were evident (all *p* > 0.05). In premenopausal women, estradiol levels positively predicted the competition for attention (β = 2.103, *p* = 0.022). In postmenopausal women, self-esteem predicted the tendency to compete overall (β = −0.208, *p* < 0.001), on appearance (β = −0.061, *p* = 0.01), on competence (β = −0.087, *p* < 0.001), and on attention/interpersonal success (β = −0.060, *p* = 0.01).

**Discussion:** These results, though cross-sectional, suggest that women continue to compete intrasexually in postmenopause, giving rise to new questions about the function of female ISC. If confirmed, the findings will indicate that hormones guide competitiveness in fertile women, whereas self-esteem accounts for individual differences in competitiveness post-reproduction. Particularly the function of postmenopausal ISC warrants further investigation.

## Introduction

Female intrasexual competition (ISC) describes the rivalry between heterosexual women over men with social status, a “good gene pool,” and resources to provide for the family (1). To outperform other women and render themselves the most desirable, women exhibit behaviors such as competitor derogation, self-promotion, self-enhancement, or other strategies to socially exclude female competitors (2, 3). Importantly, ISC describes behaviors which are primarily applied to enhance one's ability to outcompete other women, and not to impress or attract men, demonstrating that mating goals can influence women's behaviors. As such, female ISC represents a unique form of social interaction. However, the exact source of female–female competition is still not fully understood.

Previous research suggests that female ISC is influenced by psychosocial and physiological, particularly endocrinological, factors. Studies have shown more pronounced ISC in young or midlife women with certain personality characteristics, including those who are less agreeable (4, 5), less extraverted, less conscientious (6), or higher in neuroticism (4, 6); findings for self-esteem are mixed (7, 8). Such findings indicate a trait-like component of ISC, explaining why certain women compete more than others. There is additional evidence that ISC fluctuates within a woman, potentially paralleled by the changing sex hormone environment across the menstrual cycle (6, 9). Framed according to the *ovulatory competition hypothesis*, the assumption is that ISC is more likely to translate into reproductive success when applied closer to ovulation (10). Although personality and hormonal factors most likely interact to predict female ISC, to the best of our knowledge there is no study investigating both factors in parallel in order to link theories from social psychology and biology.

Women at the end of the reproductive lifespan represent the ideal study population, as they allow for a controlled hormonal environment to study the contributions of hormones and personality characteristics to ISC. At an average age of 51 years, women reach menopause, a state characterized by relatively low and stable sex hormones, including estradiol, progesterone, testosterone, and dehydroepiandrosterone sulfate (DHEAS) (11, 12). To date, only two studies have explicitly tested a menopausal effect of female ISC. The first study, by Vukovic (13), examined women aged 40 to 64 years, with participants being asked to rate their preference for either feminized or masculinized facial photographs of young adult men and women. Those who had not had a menstrual cycle in at least 12 months (i.e., postmenopausal women) revealed a stronger preference for femininity in faces of young women than did women who still had menstrual cycles (i.e., pre- and perimenopausal women). At the same time, there was no effect of menopausal stage on preferences for male facial photographs. The results could not be explained by the participants' age, and thus indicate a true menopausal effect, in which the aversion to young, attractive females seems to decrease in post-reproductive women (13). Moreover, the findings were replicated in a study from the same workgroup using a similar study design, but with facial photographs of men and women of the participants' age (14). Although these findings provided critical insights into the source of ISC, both studies used fixation times on other females' faces as an indicator of ISC, and therefore do not yield any evidence on the general tendency to perceive interactions with other women as competition. Furthermore, the studies did not assess personality characteristics and additionally had some methodological limitations, including the lack of control for the menstrual cycle phase in the younger group and no assessment of continuous sex hormone levels as indicators of fertility.

To summarize, female ISC seems to be influenced by an interaction of personality characteristics and sex hormones, although no study so far has investigated both in parallel. Moreover, there is some indication of an overall decrease in competitor derogation in the post-reproductive phase, while no study has investigated the menopausal effect on the general tendency to perceive an interaction with another woman as competition. Therefore, we sought to contribute to the scarce literature on the menopausal effect of female ISC by investigating the effect of menopausal stage and continuous sex hormone levels on the general tendency to compete intrasexually. The second aim was to test, for the first time in parallel, the contributions of sex hormones and personality characteristics to female ISC.

## Materials and Methods

The present analyses are part of the Women 40+ Healthy Aging Study (15). This cross-sectional study was conducted at the University of Zurich and targeted subjectively healthy community-dwelling women between the ages of 40 and 75 years to study biopsychosocial contributors to healthy aging.

### The Current Study

Participants were recruited via flyers, Facebook, articles in health-related online portals, newsletters, and mailing lists. Women were included in the study if they reported to have at least good self-rated health (16) and fulfilled the defined inclusion criteria. These criteria were assessed first in an online self-screening and additionally confirmed by a trained study member in a telephone screening. Women were not included if they had any acute or chronic somatic disease or mental disorder, had received psychotherapeutic or psychopharmacological treatment during the last 6 months, or consumed more than two standard units of alcohol per day. Additional exclusion criteria included a pregnancy in the last 6 months, premature menopause or a surgical menopausal status due to the removal of either both ovaries or the uterus, routine use of any kind of hormonal medication including oral contraceptives or hormone therapy in the last 6 months, employment on shift work, or recent long-distance flight travel.

### Study Procedures

Participants were invited to our laboratory between June 2017 and February 2018. All sessions were conducted on a weekday. Participants were instructed to avoid physical exercise for at least 24 h before their laboratory visit and to abstain from eating or drinking (except for water) on this day. Sessions all started at 7.45 a.m., and saliva samples were taken at 8.00 a.m. On the same day, participants completed an online survey including validated measures for ISC and personality characteristics. For premenopausal women, the sessions were timed according to their bleeding patterns and all took place in the follicular phase of the menstrual cycle (after bleeding cessation). A counting method was used to ensure testing in the follicular phase, starting on the day of menstruation (when women informed the study team) and adding the woman's average length of bleeding to schedule the appointment in the early or mid-follicular phase in order to avoid testing at the time of ovulation.

Menopausal stage was assessed according to the standard Stages of Reproductive Aging Workshop (STRAW + 10) criteria (17) and women were described as postmenopausal if they had not had a menstrual cycle in the last 12 months. Of the 130 women who completed the entire study, 10 women were perimenopausal (irregularity in the length of consecutive cycles or skipped cycles; 17). These women were excluded from the current analyses, as perimenopause is characterized by drastic day-to-day sex hormone variability (18). Moreover, eight women reported to be bisexual (*n* = 6), homosexual (*n* = 1), or asexual (*n* = 1) and were excluded from the present analyses as these samples were too small to explicitly test for the effects of sexual orientation on female ISC. Three participants did not report on ISC and were therefore excluded from further analyses. The final sample therefore consisted of 109 women.

The study was conducted in accordance with the recommendations of the Cantonal Ethics Committee (KEK) Zurich, which reviewed and approved the protocol. All subjects gave written informed consent to participate in this study in accordance with the Declaration of Helsinki.

### Intrasexual Competition

The validated German version of Buunk and Fisher's (5) Scale for Intrasexual Competition was applied (5, 6). The questionnaire consists of 12 items which are rated on a 7-point Likert scale ranging from (1) not at all applicable to (7) completely applicable (i.e., “I can't stand it when I meet another woman who is more attractive than I am.” “I tend to look for negative characteristics in attractive women.”). Overall scores can range from 12 to 84 points. The Cronbach's alpha in this study was 0.86, suggesting a good internal consistency. As proposed in our validation study, subscales were calculated for the competition on (I) attention and interpersonal success, (II) competence, and (III) appearance (6). Cronbach's alphas for these subscales were 0.80 for attention and interpersonal success, 0.79 for competence, and 0.75 for appearance. All subscale scores can range from 4 to 28 points.

### Big Five Personality Traits

The German version of the Big Five Inventory short version was used [BFI-K; (19)]. This questionnaire consists of 21 items rated on a 5-point Likert scale ranging from (1) not at all applicable to (5) very applicable. The Cronbach's alphas in this study were 0.80 for extraversion, 0.59 for agreeableness, 0.59 for conscientiousness, 0.74 for neuroticism, and 0.71 for openness, which are comparable to the values reported in the validation study (19).

### Self-Esteem

The German-language Multidimensional Self-Esteem Scale [MSWS; (20)] was used to assess the overall self-esteem. The global self-esteem score consists of 32 items rated on a 7-point Likert scale ranging from (1) not at all to (7) very much. The items cover emotional and achievement-related self-esteem, social skills, social confidence, physical attractiveness, and sportiness. The Cronbach's alpha of 0.75 in the present study suggests an acceptable internal consistency.

### Sex Hormones

Saliva samples were collected in 2-ml SaliCaps (IBL International GmbH, Hamburg, Germany) using the passive drool method and were subsequently stored at −20°C. At the end of data collection, saliva samples were thawed, centrifuged, and analyzed for estradiol (pmol/L), progesterone (pmol/L), DHEAS (pmol/L) and testosterone (pmol/L) using enzyme-linked immunosorbent assays (ELISA, IBL International GmbH, Hamburg, Germany). All salivary analyses were performed at the Biochemical Laboratory at the Psychological Institute of the University of Zurich. Intra- and inter-assay variation was <10%, with a sensitivity of 1.10 pmol/L for estradiol, 8.24 pmol/L for progesterone, 0.05 ng/ml for DHEAS, and 6.25 pmol/L for testosterone.

### Power Calculations

Since the present analyses are part of a larger study powered to detect the effect of biopsychosocial predictors on female healthy aging, analyses for the present sample were calculated as sensitivity analyses. With a sample size of 109 (n1 = 53, n2 = 56), assuming a power of 80 % and a two-sided alpha level of 0.05, the study was powered to detect medium-sized effects between pre- and postmenopausal women's ISC, and small-sized effects of continuous hormones and personality factors on ISC.

### Statistical Analyses

Three participants had one missing item on the *Scale for Intrasexual Competition*, which was replaced with the mean of the other 11 items. Hormone levels at three standard deviations from the mean were winsorized. Distributions of hormone levels were visually inspected, and since all hormones were highly left-skewed, a log-transformation was applied. Next, *t*-tests were run to test for differences in ISC (overall score and subscales) between pre- and postmenopausal women. Finally, separate multiple linear regression models were run to test for an effect of continuous hormone levels and personality characteristics on ISC. Further models were run to test for an interaction of predictors with menopausal stage. Analyses were performed using SAS (SAS Institute Inc; Cary, NC.). Two-sided *p*-values at <0.05 were considered as statistically significant.

## Results

A total of N = 109 healthy female participants were included in the analyses. Premenopausal women were significantly younger than postmenopausal women (mean = 45.57, standard deviation (SD) = 3.72 vs. mean = 59.91, SD = 6.62, *t*_(107)_ = −13.84, *p* < 0.001). Table 1 summarizes the sample characteristics and Table 2 depicts the levels and distributions of sex hormones and personality characteristics for pre- and postmenopausal women separately.

**Table 1:** Demographic characteristics.

Education *n* (%)	
Vocational training	40 (36.7)
College or vocational college degree	16 (14.6)
Bachelor/Master/Ph.D.	47 (43.1)
Other	6 (5.5)
Employment status *n* (%)	
Working 10–60%	33 (30.2)
Working 61–100%	54 (49.5)
Unemployed	3 (2.8)
Retired	14 (12.8)
Working in own household	5 (4.6)
Marital status *n* (%)	
Married	62 (56.9)
Single	25 (22.9)
Divorced	17 (15.6)
Widowed	5 (4.6)
Years since final menstrual period	9.26 (1–25)
(for postmenopausal women) mean (range)	

**Table 2:** Descriptive statistics for pre- and postmenopausal women.

**Parameter**	**Premenopausal women** **(*N* = 53)**	**Postmenopausal women** **(*N* = 56)**
	**Mean (SD; range)**	**Mean (SD; range)**
Intrasexual competition	25.46 (9.28; 12–55)	25.75 (10.88; 12–64)
Appearance	10.49 (4.23; 4–19)	9.73 (4.51; 4–20)
Competence	6.72 (2.90; 4–18)	7.82 (4.10; 4–22)
Attention/interpersonal success	8.25 (4.26; 4–22)	8.20 (4.42; 4–23)
Big five personality traits		
Self-esteem	167.19 (22.02; 105–215)	167.50 (25.20; 110–214)
Extraversion	14.50 (3.38; 7–20)	14.39 (3.35; 7–20)
Agreeableness	13.38 (2.92; 8–19)	13.70 (2.76; 8–20)
Conscientiousness	16.68 (2.30; 10–20)	16.41 (2.12; 11–20)
Neuroticism	10.74 (3.03; 4–17)	10.27 (2.86; 5–16)
Openness	18.91 (3.03; 11–25)	20.25 (3.23; 10–25)
Sex-hormones		
Estradiol (pmol/L)	9.09 (6.11; 1.3–25.7)	3.54 (2.65; 0.6–14.8)
Progesterone (pmol/L)	130.63 (135.80; 24.6–588.8)	94.52 (87.79; 3.6–420.2)
Testosterone (pmol/L)	38.2 (30.38; 4.9–124.7)	29.44 (25.35; 3.6–116.7)
Dehydroepiandrosterone	4.32 (2.27; 1.4–12.1)	3.82 (2.74; 1.20–13.6)
-sulfate (ng/ml)		

*SD, standard deviation. Sex hormone levels represent untransformed values*.

### Association of Menopausal Stage With Intrasexual Competition

Comparisons between premenopausal and postmenopausal women revealed no significant group differences in the ISC overall score, or in the subscales regarding competition on appearance, competence, and attention/interpersonal success (all *p* > 0.05, see Table 2 for group mean levels).

### Association of Single Hormone Levels With Intrasexual Competition

Linear regression models indicated no main effect of continuous hormone levels on ISC (overall score and subscales; all *p* > 0.05). Subsequent *post hoc* analyses testing for the interaction with menopausal stage showed that estradiol was a significant predictor of the competition for attention in premenopausal women. Specifically, premenopausal women with higher estradiol levels showed higher competition for attention compared to premenopausal women with lower levels (β = 2.103, SEM = 0.891, *p* = 0.022).

### Association of Personality Characteristics With Intrasexual Competition

**Figure 1:**
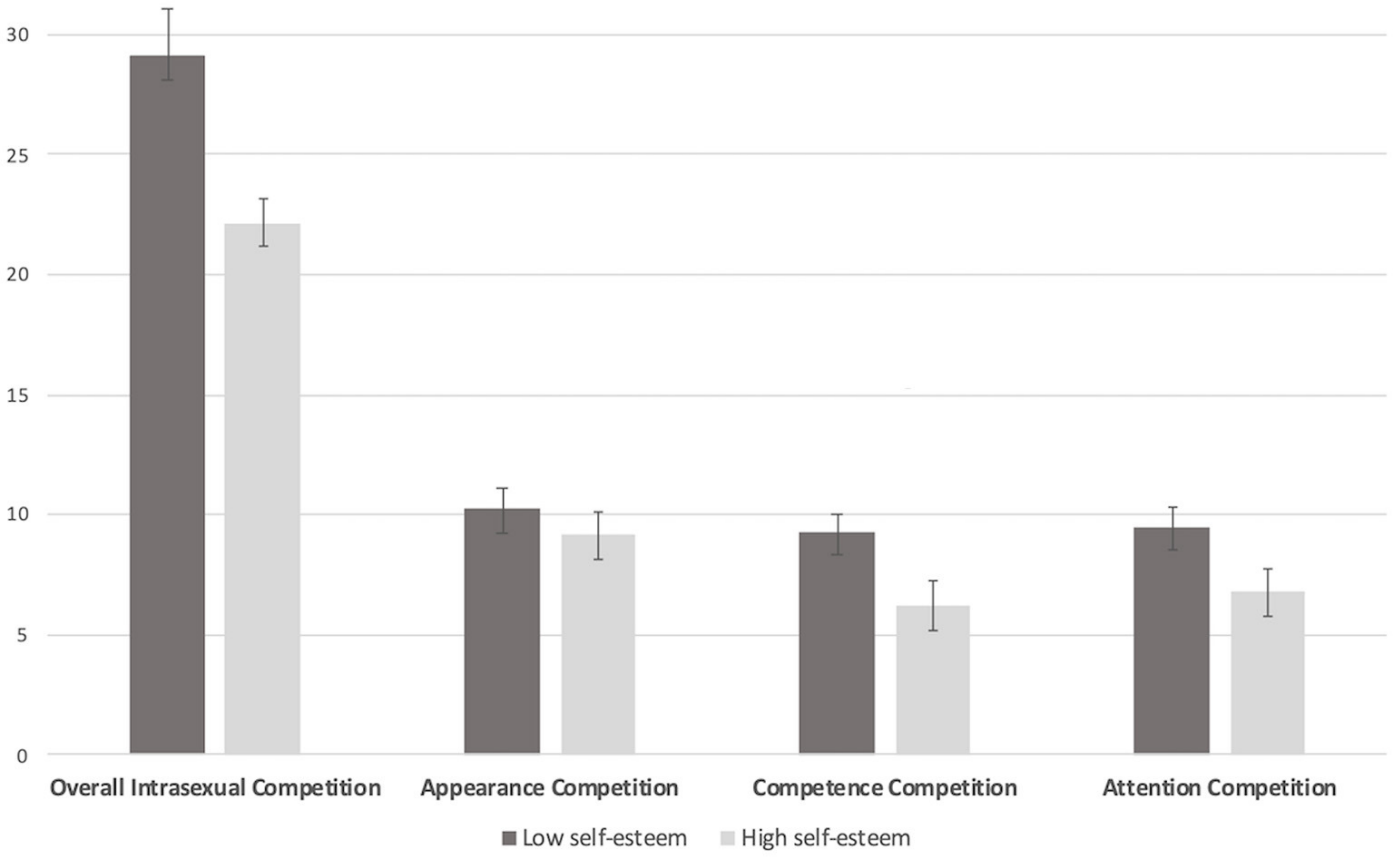
Model-based estimates of the relationship between intrasexual competition (overall score and subscales of the scale for intrasexual competition) and self-esteem in postmenopausal women.

Self-esteem was a significant predictor of all facets of ISC. Despite similar levels of self-esteem in pre- and postmenopausal women (mean = 167.2, SD = 22.02 vs. mean = 167.5, SD = 25.20, *t*_(107)_ = −0.07, *p* = 0.95), *post hoc* analyses testing for the interaction of self-esteem and menopausal stage showed that the effect was only significant in postmenopausal women. Specifically, postmenopausal women with lower self-esteem had a higher tendency to compete overall (β = −0.208, SEM = 0.051, *p* < 0.001), on appearance (β = −0.061, SEM =.023, *p* = 0.01), on competence (β = −0.087, SEM =.019, *p* < 0.001), and on attention/interpersonal success (β = −0.060, SEM =.022, *p* = 0.01). Figure 1 depicts the difference in ISC between postmenopausal women with high compared to low self-esteem. A median split was performed for descriptive purposes only.

## Discussion

In this study, pre- and postmenopausal women did not differ in their tendency to compete intrasexually and there was no evidence that continuous hormone levels predicted the overall amount of ISC. For premenopausal women, the tendency to compete on appearance was more pronounced in women with higher estradiol levels. For postmenopausal women, lower self-esteem was predictive of a higher overall amount of competition and a higher tendency to compete on appearance, competence, and attention/interpersonal success. The present study was the first to investigate hormonal and personality contributors to ISC in parallel and the first to demonstrate that overall, women seem to remain just as competitive after reproductive senescence as premenopausal women in the follicular phase.

The primary aim of the study was to investigate the contribution of sex hormones and personality characteristics to female ISC in order to better understand the source of competitive behavior among women. We expected to find clear differences in the tendency to compete intrasexually between pre- and postmenopausal women. This assumption was based on the evolutionary hypothesis stating that female ISC is a reproductive success-driven phenomenon, and on the two existing studies showing lower competitor derogation in postmenopausal women (13, 14). Overall, pre- and postmenopausal women in the present study did not differ in terms of their ISC. However, the predictors of competition varied according to the menopausal stage, with estradiol predicting premenopausal ISC and self-esteem predicting postmenopausal ISC. We interpret these findings as a shift in the reasons to compete intrasexually, from reproductive-driven in premenopause to potentially more self-esteem-driven in postmenopause. This finding is in line with a recent meta-analytic finding showing that whereas the ISC domains vary across a woman's lifespan, the overall tendency to compete only shows a marginal change [0.001 decrease in ISC score with every additional life year; (21)].

For premenopausal women, the competition on appearance was more pronounced in women with higher estradiol levels, a finding which at least partially confirms the ovulatory competition hypothesis. Estradiol shows a linear increase in the follicular phase of the menstrual cycle, whereas the other investigated hormones (progesterone, DHEAS, testosterone) are all relatively stable during this cycle phase. Therefore, this finding is in line with the follicular phase hormonal profile and corresponds to previous studies demonstrating more pronounced competition during high estradiol windows [i.e., (21, 22)]. Interestingly, estradiol was only predictive of the competition on appearance, and did not predict the other forms of competition that we assessed. This finding might indicate a hormone-specific effect on different subsets of competition, some of which might be related to cyclical changes in sex hormones (3). Researchers interested in sex hormone contributions to female ISC in reproductive-age women are therefore encouraged to investigate the effect of cyclical sex hormone changes on the differential subsets of competition, including those captured by the scale used in this study. The study by Hahn (9) provides a good framework to study within-person changes in ISC across the menstrual cycle.

This is one of only three studies to include postmenopausal women in ISC research and the first to suggest an effect of self-esteem, which therefore needs to be discussed based on the more general literature. Self-esteem describes the appraisal of one's own value (cognitive self-evaluative component) and the way one feels about oneself (affective component) (23). Although for most women, self-esteem seems to be unaffected by menopause [i.e., (23, 24)], the transition to the post-reproductive stage might still pose a threat to self-esteem in some women (25). This might include women who have a more negative attitude toward menopause and toward being a woman who is no longer capable of bearing children. It can be hypothesized that for women who identify strongly with gender norms around being a childbearing woman, transitioning to menopause could signify the loss of an important part of their identity. This experience of loss might be even more pronounced in women with lower self-esteem, who might already have fewer aspects in life that can provide a sense of self-worth. To protect their self-esteem, these women might feel pressured to outperform other women whom they perceive as a potential threat, in order to prove to themselves and others that they are (still) competent, attractive, and witty. It might also be the case that these women tend to avoid other women who pose a threat to their self-esteem, a part of ISC that is often overlooked but might actually be the preferred behavioral tendency in women with lower self-esteem (7). Either way, women with lower self-esteem might be more sensitive to detecting actual or perceived cues that prove their negative self-concept, thus increasing their tendency to compete intrasexually. Such hypotheses will need to be tested in future studies, preferably with a combination of self-report measures of ISC, self-esteem, identification with gender roles, and attitudes toward menopause, along with behavioral observations of competition.

## Strength and Limitations

More than 30 years ago, it was first empirically demonstrated that women compete intrasexually (1), but the literature on female ISC remains scarce. As such, this study provides an important contribution to the limited knowledge on female ISC in general and on post-reproductive competition specifically. The present design was more methodologically sound than the two comparable studies (13, 14), as it only included pre- and postmenopausal women, thus excluding perimenopausal women, and timed assessments in the premenopausal women to the follicular phase of the menstrual cycle. Moreover, none of the investigated women were using any hormonal contraceptive, hormone replacement, or any other medication influencing the endocrine system, which reduced the risk of exogenous factors confounding our outcomes. Well-validated self-report measures were applied to quantify the general tendency to compete intrasexually and to assess personality characteristics.

ISC was measured using a questionnaire and we did not invoke a direct comparison or activate the tendency to compete. Although this does not constitute a general limitation, it does limit the ability to compare our results with the previous findings of menopausal effects on female ISC. While the *Scale for Intrasexual Competition* is a widely used instrument which was validated in women, this scale might include items that are not suitable for some women, such as retired women (i.e., “*I would not hire a very attractive woman as a colleague*”). In addition, the questionnaire does not assess the subjective thought processes of each participant in order to rate their competitiveness. For instance, some women might have envisioned a younger or more attractive competitor while completing the questionnaire, whereas others might have imagined themselves in situations with same-age or even older competitors. This is certainly an advantage of study designs that intentionally invoke competition in the lab. For postmenopausal women, there was a relatively large range of years since women had reached their final menstrual period (FMP). Years since the FMP were, however, not significantly correlated with ISC.

The cross-sectional nature of this study must be noted as an additional limitation. Hormones were only measured once, and therefore no conclusions can be drawn about the contribution of hormonal changes to ISC in premenopausal women. The decision to investigate hormones and ISC in the follicular phase in premenopausal women might have limited the power to detect a menopausal effect. Potentially, comparisons with women in the ovulatory phase would have resulted in stronger effects. As such, our findings will need to be replicated in a longitudinal study including several hormonal assessments at (1) different menstrual cycle phases per individual, (2) following women in the transition from the pre-, to peri-, to postmenopausal stage, or (3) in comparison to a group of women of the same age, but who have undergone surgical menopause. In this way, possible confounding between-group aspects including a generation effect could be ruled out. Although the in our study, ISC was independent of the number of children or the satisfaction with the choice to have had or have not had children (results not reported), this may be the case in samples which are less satisfied with their family choices than the participants in this present healthy aging study (26). Finally, we investigated a population of highly educated, heterosexual women with a high socioeconomic status and high levels of well-being, thus limiting the generalizability of these results to women of different backgrounds or sexual orientations.

## Conclusions

This study adds to the scarce literature on a menopausal effect of ISC and gives rise to new questions about the function of female ISC. Our findings suggest a shift from reproductive-driven ISC in premenopause to potentially more self-esteem-driven ISC in postmenopause. In particular, the function of postmenopausal ISC warrants further investigation.

## Data Availability Statement

The raw data supporting the conclusions of this article will be made available by the authors, without undue reservation.

## Ethics Statement

The studies involving human participants were reviewed and approved by Cantonal Ethics Commission (KEK) Zurich. The patients/participants provided their written informed consent to participate in this study.

## Author Contributions

SF, LM, and UE were responsible for the design of the study. SF and CA performed the analyses and drafted the background, methods, results, and discussion sections. All authors contributed to the discussion of the results and approved the final version of the manuscript.

## Conflict of Interest

The authors declare that the research was conducted in the absence of any commercial or financial relationships that could be construed as a potential conflict of interest.

## Publisher's Note

All claims expressed in this article are solely those of the authors and do not necessarily represent those of their affiliated organizations, or those of the publisher, the editors and the reviewers. Any product that may be evaluated in this article, or claim that may be made by its manufacturer, is not guaranteed or endorsed by the publisher.
